# Costing curative outpatient care for the poorest in Burkina Faso: informing universal health coverage and leaving no one behind

**DOI:** 10.1186/s12913-024-11854-8

**Published:** 2024-11-28

**Authors:** Yvonne Beaugé, Valéry Ridde, Sidibé Souleymane, Joël Arthur Kiendrébéogo, Hoa Thi Nguyen, Emmanuel Bonnet, Manuela De Allegri

**Affiliations:** 1 Heidelberg Institute of Global Health, Heidelberg University Hospital and Medical Faculty, 69120 Heidelberg, Germany; 2 Medical Faculty, Institute for Medical Education and Clinical Simulation, Goethe University Frankfurt, Frankfurt, 60590 Germany; 3 CEPED, IRD- Université Paris Cité, ERL INSERM SAGESUD, Paris, France; 4 Ecole Doctorale de Science et Santé, LASAP, IRD (French Institute for Research on sustainable Development), AGIR, Université Joseph KI-ZERBO, Ouagadougou, Burkina Faso; 5 Department of Public Health, Health Sciences Training and Research Unit, University Joseph Ki-Zerbo, Ouagadougou, Burkina Faso; 6 French Institute for Research on Sustainable Development (IRD), UMR 215 Prodig, 5, cours des Humanités, F-93 322, Aubervilliers Cedex, France

**Keywords:** Poorest, Universal Health Coverage, Cost Analysis, Burkina Faso, Curative Outpatient Care, Budget Impact Analysis

## Abstract

**Introduction:**

The poorest in Burkina Faso face numerous barriers to healthcare access, including financial and geographic obstacles, as well as a high burden of chronic conditions and multimorbidity. This study estimates the average cost of providing curative outpatient consultations at first-level healthcare facilities to the poorest in Burkina Faso. It also estimates the budgetary impact of scaling up free access to these services nationwide. The findings provide essential evidence on cost structures to inform decision-makers in developing policies aimed at achieving universal health coverage and ensuring that no one is left behind.

**Methods:**

We conducted a micro-costing study to estimate the economic costs of providing curative outpatient healthcare services to the poorest at first-level healthcare facilities, considering a health system perspective. We measured the consumption of capital costs (building and equipment) using survey data from 32 primary health facilities and recurrent costs (drugs and consumables) from medical records of 1380 poor patients in Diébougou district. These individuals were targeted and exempted from user fees through a community-based targeting approach. We obtained unit costs from official price lists, pharmacy registries, and expert interviews. We calculated the national budget for providing curative care services to the exempted poorest based on the average cost per first-level consultation.

**Results:**

The estimated capital and recurrent costs of providing curative care services ranged between USD 0.59 - USD 0.61 and USD 2.58 - USD 5.00, respectively. The total cost ranged between USD 3.17 - USD 5.61 per first-level consultation. Providing curative care to the bottom 20% of the population, assuming 0.25 healthcare contacts per person per year, would result in an annual expense ranging from USD 2.77 M to USD 5.38 M (0.74-1.43% of the healthcare budget in 2019). With 2 healthcare contacts per person per year, costs increase to USD 22.19 M to USD 43.05 M (5.91-11.45% of the healthcare budget).

**Conclusion:**

The results can inform policies aimed at expanding access to curative care for the poorest in Burkina Faso, contributing to the goals of universal health coverage and leaving no one behind. Further research is needed to enhance cost estimation and budgeting for higher-level care in the country.

**Supplementary Information:**

The online version contains supplementary material available at 10.1186/s12913-024-11854-8.

## Introduction

Universal Health Coverage (UHC), a critical component of the United Nations’ third Sustainable Development Goal, aims to provide equitable access to quality health services for everyone without financial hardship [1]. Despite global efforts to expand access to healthcare, over 400 million poor and vulnerable people in low- and lower-middle-income countries (LMICs) [2] still face persistent and severe health disparities due to limited access to essential health services [3]. To ensure that existing inequities are taken into account in the design of health policies, UHC embraces the Leaving No One Behind (LNOB) principle [4] prioritizing the expansion and improvement of effective service delivery models for the poorest.

In LMICs, providing quality healthcare services to the poorest populations is challenging due to their remote locations, limited health literacy, and complex health needs [5]. As a result, delivering healthcare services to this group may involve higher costs compared with the general population. Still, there is a lack of accurate costing studies addressing the specific needs of the poorest individuals [6]. In turn, this lack of accurate cost information represents a real barrier to the design and implementation of programs aimed at enhancing access to care for this specific segment of the population, such as targeted publicly-funded health insurance schemes or free health care policies. Existing literature on the costs of healthcare services in LMICs mainly focuses on the general population, specific diseases, or other specific populations [7–10], leaving a significant research gap regarding the costs of services for the poorest individuals.

Burkina Faso, a country committed to improving healthcare access for its poorest populations, has implemented various health financing reforms, including community or occupational based health insurance and user fee removal for pregnant women and children under the age of five [11]. Notably, the Régime d’Assurance Maladie Universelle scheme (RAMU), stands out as a national health insurance program enacted into law on 5th September 2015 [12] to provide quality health services to all, with a particular emphasis on the poorest and most vulnerable populations. By aligning with the policy objectives of UHC and LNOB, Burkina Faso aims to eliminate financial barriers and enhance health outcomes for all its citizens.

However, the aforementioned challenges persist in effectively targeting and extending coverage to all poor and informal workers [6, 12], necessitating a closer examination of the economic costs associated with providing curative outpatient care to the poorest population in Burkina Faso at first-level healthcare facilities. Therefore, we conducted a comprehensive micro-costing study adopting a health system perspective to provide decision-makers with crucial cost data to inform the budgeting and implementation of policies to facilitate free access to care for the poorest.

## Methods

### Study setting

Burkina Faso is located in West Africa and had a population of 21.51 million in 2021. The country is positioned among the world’s poorest nations. Over 40% of its population lives in poverty, surviving on less than USD 1.90 a day [13]. This study specifically examines the so called ‘ultra-poor’ [14], who are in an advanced state of poverty and lack basic necessities, such as food, shelter, and sanitation, as well as the financial and social resources necessary to access and pay for essential healthcare services [15–17]. To enhance readability, the term ‘ultra-poor’ is interchangeably referred to as the ‘poorest’ throughout this manuscript.

The study is conducted in the Diébougou health district in Bougouriba Province, South-West Burkina Faso. The district has a population of 139,824 [18]. The district presents a unique setting for this study due to the introduction of the Performance-based Financing (PBF) intervention, combined with a community-based targeting and exemption mechanism for the poorest in 2016, provided a valuable opportunity to distinguish medical records of the poorest from non-poor patients [19]. The PBF initiative not only aimed to improve healthcare quality and accessibility, but also introduced mechanisms to identify and exempt the poorest from health care payments [19], the key population of this study.

Diébougou district is served by 24 government healthcare facilities, including 4 dispensaries, 19 Primary Healthcare Facilities (Centre de Santé et de Promotion Sociale - CSPS), and one district hospital (Centre Médical avec Antenne Chirurgicale - CMA). The healthcare infrastructure includes eight general practitioners and two pharmacists [20]. Across Burkina Faso, primary level healthcare services are provided at the CSPS and the CMA, with each CSPS serving approximately 8,000–15,000 individuals and covering 5 to 23 villages. This study focuses on first-level services offered by CSPS, primarily located in the rural and peripheral areas of Burkina Faso [21].

### Study design and overall approach

To provide policy makers with relevant cost information, we adopted a health system perspective and employed two different approaches to address our study objectives. Firstly, we conducted a micro-costing study using a bottom-up approach to estimate the average cost of providing a curative outpatient consultation at the CSPS level (first-level of services) to the exempted poorest. Curative outpatient care refers to healthcare services provided to individuals who visit the healthcare facility for the diagnosis and treatment of either acute or chronic diseases or conditions without requiring hospital admission. The ability to distinguish medical records of the poorest from non-poor patients was enabled by the prior implementation of the PBF intervention in the Diébougou district in 2016.

Secondly, we used the estimated cost per consultation to assess the budget impact of providing first-level curative outpatient care to the poorest across the country.

The base year of the cost analysis is 2019, in order to align with the research project’s deliverables. To account for inflation, we adjusted all costs incurred before 2019 using the national consumer price index (CPI) [22]. We multiplied the cost incurred in 2016 by the ratio of the relevant CPI (CPI 2019 = 108.36 / CPI 2016 = 108.23). We obtained Burkina Faso’s annual CPI from the International Monetary Fund, International Financial Statistics [23]. We converted values from FCFA to USD using the average exchange rate for 2019 (1 USD = FCFA 585.91) [24]. We used Microsoft Excel 2019 (Microsoft, Redmond, WA) to operationalize the model.

### Identification of cost categories and measurement of resource consumption

We identified two cost categories for the study: capital costs and recurrent costs. Green (1999) and Creese (1994) define capital costs as one-off expenditure or inputs that lasts more than one year [25, 26]. Recurrent costs are the costs of maintaining and operating a given program once the initial, one-off investment has been completed [27]. Capital costs included building and equipment costs, while recurrent costs included consultation fees, drugs, and human resources. Local experts were consulted to identify these cost categories. The quantity of rapid tests and the use of test strips was minimal and thus excluded. Resource consumption for recurrent costs and capital costs was measured using patient registries and a health facility survey, respectively.

Patient registries from 15 CSPS in the Diébougou district were used from January to December 2016 in order to estimate the recurrent resources consumed by the poorest. Ten enumerators collected the paper-based information and transferred it to Excel. In total, 1380 patient records were used after excluding children below five years (who were covered by the national free health care policy for the pregnant women and children under five) and records with missing values. We conducted an additional data collection in August 2020 to obtain unit cost information for the drugs used in the medical records. We created a drug list matrix based on the entries in the records, and employing a trained enumerator to collect the unit cost information from pharmacy registries (Supplementary file 1). Drug prices have remained consistent over the years in the study area, according to the pharmacists.

For capital costs, we utilized a health facility survey (Supplementary file 2 and 3) conducted between March and May 2018 as part of another research project, which collected information on capital costs and variable overheads from 32 CSPS distributed across four regions (North, Hauts-Bassins, Est, and Centre South) by 20 trained enumerators. Paper-based responses were transferred to Excel. We extracted information on equipment for this study.

### Valuation of costs

We used a standardized approach to value costs by relating the unit cost of each resource to the quantity measured in the previous step. We obtained unit cost information from various sources. Recurrent costs were estimated from the fee structure of healthcare facilities for consultation services, while average drug prices among adults were derived from pharmacy registries. For human resource costs not covered by the consultation fees, we used the human capital approach, extracting salary information for nurses, midwives, and mobile health workers from data collected within the PBF end-line impact evaluation framework in the Diébougou district. The salaries of doctors and pharmacists were adopted from literature [28], based on the average gross monthly salary, with a working month consisting of 22 working days and eight hours/day.

To estimate building costs, we interviewed Ministry of Health experts to determine the average cost of building a CSPS (104 million FCFA without equipment) and the useful lifespan of the CSPS (25 years). We derived the average size of a CSPS (500 sq m) from construction plans and used the square meters occupied for consultations (245 sq m) from the construction plan to allocate the building cost for consultation service (104 million FCFA * 245/500). For equipment, we estimated the unit costs and useful lifespan by triangulating information from two ministry structures: *Direction des Infrastructures*,* de l’Equipement* et de la Maintenance and *Société de Gestion de l’Equipement et de la Maintenance Biomédicale* - Management Company of Biomedical Equipment and Maintenance (SOGEMAB). Straight-line depreciation was applied by dividing the item’s value by its useful lifespan. For equipment with a defined value below FCFA 15,000 (USD 25.50), we used the approach taken by Flessa & Marshall (2009) [29] and depreciated them within a year.

### Cost data analysis

To analyze the cost of a first-level curative outpatient consultation, we differentiated between capital and recurrent costs. Valuing these costs involved combining information on resource consumption with the unit prices described earlier [30]. To account for indirect administrative expenses, we applied an average overhead rate of 20% to the total fixed and variable costs. This approach is consistent with the guidelines outlined in the International Standard Cost Model Manual [31].

We estimated the average building and equipment costs per consultation by dividing the total building and equipment costs by the total number of consultations. The average cost per consultation was then calculated as the sum of the average recurrent costs for each cost item (human resource, consultation, drugs, and variable overheads) and the average capital cost.

We conducted a two-way sensitivity analysis to account for the uncertainty surrounding the drug and overhead estimates. Specifically, we applied the mean drug expenditure of FCFA 657.68 plus one standard deviation (FCFA 545.36) and two standard deviations (FCFA 1,090.72). In addition, we increased the overhead rate for capital and recurrent costs from 20 to 25%.

This approach allowed us to obtain a comprehensive understanding of the cost and to identify the key cost drivers.

### Budget impact analysis (BIA)

We conducted a BIA to estimate the financial impact of providing first-level curative healthcare services to poorest nationwide on the Burkinabe healthcare budget. For this analysis we focused only on recurrent costs.

If the government of Burkina Faso were to provide first-level curative services for the poorest without charging user fees, they would need to plan a budget to cover the recurrent costs for the health facilities.

**Fig. 1:**
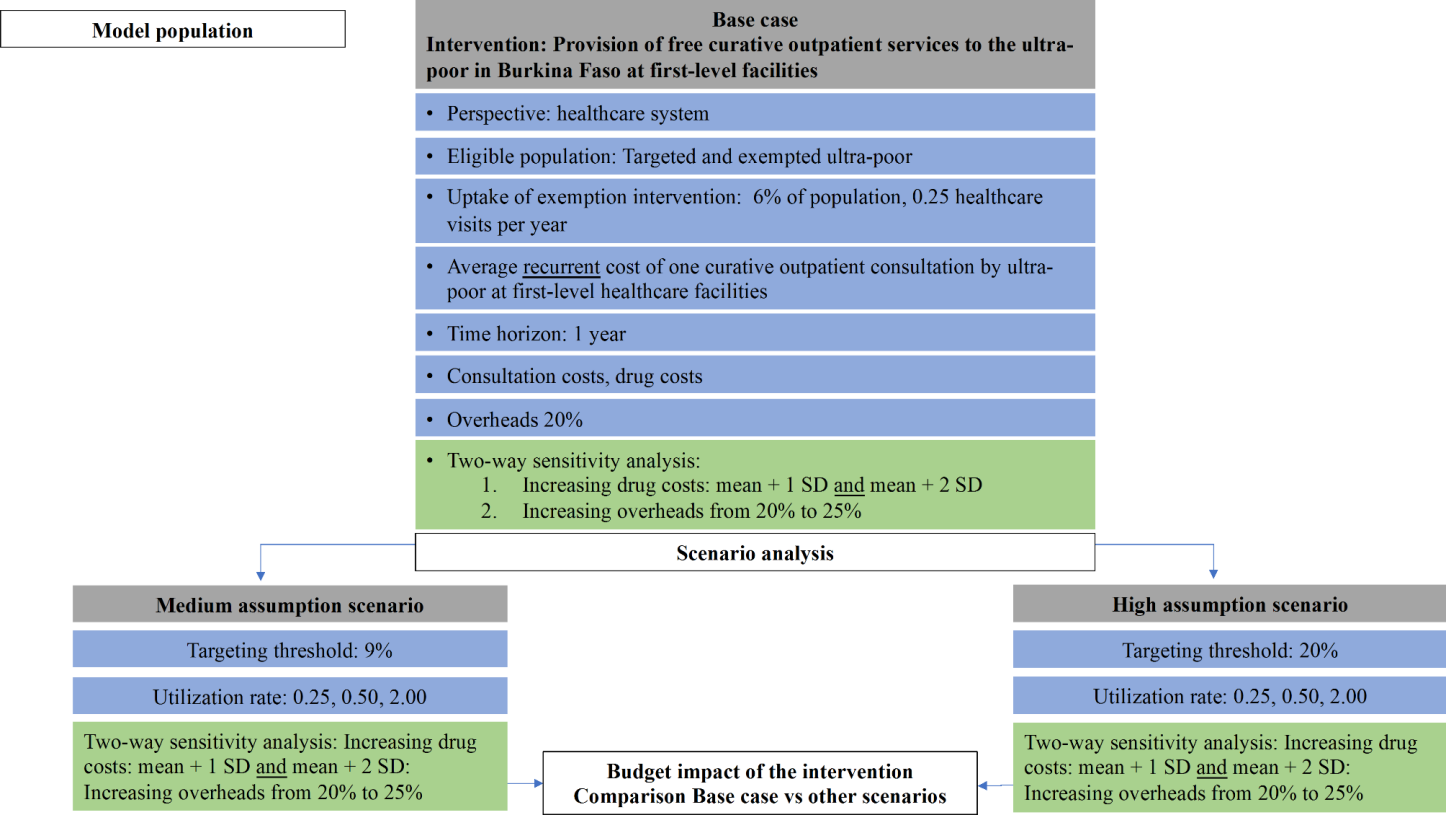
Conceptual framework for BIA. This figure illustrates the conceptual framework developed for the BIA of providing first−level curative outpatient healthcare services to the poorest in Burkina Faso. The scenario names are shown in dark grey, and the green box indicates where each scenario differs from the base case. The white box indicates the economic endpoints. Utilization rates were estimated based on data from the poorest in Diébougou district, with an average of 0.25 healthcare contacts per person per year in the base case, as determined by the authors. This framework allowed us to assess the financial impact of different scenarios and identify those that would have the greatest impact on the healthcare budget

To guide our analysis, we developed a model framework following the guidelines on BIA for healthcare interventions, depicted in (Fig. 1). We used a cost-calculator model to estimate the annual costs, which multiplies the annual service volume by its average costs.

### Model inputs

The model used four inputs:

*Eligible Population: *The model population consisted of 6%, 9%, and 20% of the total population in Burkina Faso, assumed to be eligible for targeted user fee exemptions. The 6% threshold was used as a community threshold, and the 9% threshold illustrated the extremely poor according to the United Nations Development Programme [32]. The 20% threshold reflected the concept of the “bottom 20% of the population,” the poorest income quintile, as measured by income inequality.

*Time frame: *The time span of one year was adopted, in line with the period of the national healthcare budget of Burkina Faso.

*Uptake of the intervention:* The poorest in the Diébougou district had, on average, 0.25 healthcare contacts per person per year, based on the estimates derived from above. The context-specific utilization rate was doubled and quadrupled to reflect a likely increase in the intervention uptake in the absence of a specific global recommendation for the number of outpatient care contacts per person per year.

*Costs:* The base case and scenario analysis inputs originated from the cost assessment described above.

### Model output

The primary output of interest was the total annual recurrent cost.

### Scenario analysis

We conducted medium and high assumption scenarios reflecting alternative values for drugs and overheads, while also considering different thresholds of population coverage (6%, 9%, and 20% of the population) and different utilization rates among the targeted poorest (0.25, 0.50, and 2.00 healthcare contacts per capita per person per year) (Table S1 and Table S2).

### Ethical considerations

Informed consent to participate was obtained from all participants in the study. This process was in accordance with ethical standards and was approved by the *Comité National d’Éthique pour la Recherche en Santé* in Burkina Faso (Decision No. 2019-01-004). No ethical clearance for this study was required in Germany since the study relied exclusively on secondary fully-anonymized data.

## Findings

The study estimated the average cost per first-level curative outpatient consultation for the poorest in Burkina Faso to be USD 3.17 (Table 1). Recurrent costs constituted 81.39% of the total cost, with drugs and human resources being the two largest cost drivers at 35.33% and 21.77%, respectively. Capital costs accounted for the remaining 18.61% of the total cost.

Scenario analyses assessed the impact of varying drug costs and overhead percentages on the total average cost. Scenario I, which increased the drug cost from USD 1.12 to USD 2.05 and the overhead percentage from 20 to 25%, increased the total average cost from USD 3.17 to USD 4.45. Scenario II, which increased the drug cost from USD 1.12 to USD 2.97 and the overhead percentage from 20 to 25%, further increased the total average cost from USD 3.17 to USD 5.61.

**Table 1:** The average cost of providing one first-level curative outpatient consultation to the poorest in Burkina Faso

Base calculation		Results Scenario analysis	
Time Horizon 1 Year		I	II
**I. Capital costs**	**Cost per consultation in USD**		
Building costs	0.26 USD	0.26 USD	0.26 USD
Equipment costs	0.23 USD	0.23 USD	0.23 USD
Fixed Overheads 20%	0.10 USD	0.12 USD	0.12 USD
**Total Capital Cost**	**0.59 USD**	**0.61 USD**	**0.61 USD**
**II. Recurrent costs**	**Cost per consultation in USD**		
Consultation costs	0.34 USD	0.34 USD	0.34 USD
Drug costs	1.12 USD	2.05 USD	2.97 USD
Human resource costs	0.69 USD	0.69 USD	0.69 USD
Variable overheads 20%	0.43 USD	0.76 USD	1.00 USD
**Total recurrent cost**	**2.58 USD**	**3.84 USD**	**5.00 USD**
**Grand total capital and recurrent cost per consultation**	**3.17 USD**	**4.45 USD**	**5.61 USD**

Table 2 shows the recurrent costs and budget impact of providing curative outpatient healthcare services to the poorest in Burkina Faso. Costs vary based on the population targeted and the expected number of curative contacts. For example, targeting 6% of the population with 0.25 curative contacts per capita would cost USD 832,225.81 annually, equivalent to 0.22% of the healthcare budget. Increasing the target population to 20% with 0.50 curative contacts would cost USD 5,548,172, representing 1.48% of the healthcare budget. Three scenarios are presented with different targeting thresholds and utilization rates, ranging from USD 832,225.81 to USD 22,192,688.35 representing 0.22–5.91% of the healthcare budget, respectively.

**Table 2:** Cost and budget impact estimates applying different targeting thresholds and population coverage

Cost category	Base case: Targeting threshold 6% of the population:1,290,611	% of the healthcare budget	Medium assumption scenario: Targeting threshold 9% of the population: 1,935,916	% of the healthcare budget	High assumption scenario Targeting threshold 20% of the population: 4,302,036	% of the healthcare budget
**Scenario 1 Utilization 0.25**						
**Consultation costs**	USD 109,833.69		USD 164,750.54		USD 366,112.31	
**Drug costs**	USD 361,177.91		USD 541,766.87		USD 1,203,926.37	
**Human resources**	USD 222,509.91		USD 333,764.86		USD 741,699.70	
**Variable overheads 20%**	USD 138,704.30		USD 208,056.45		USD 462,347.67	
**Total recurrent cost in USD**	**USD 832 , 225.81**	**0.22**	**USD 1 , 248 , 338.72**	**0.33**	**USD 2 , 774 , 086.04**	**0.74**
**Scenario 2: Utilization 0.50**						
**Consultation costs**	USD 219,667.38		USD 329,501.07		USD 732,224.61	
**Drug costs**	USD 722,355.82		USD 1,083,533.73		USD 2,407,852.74	
**Human resources**	USD 445,019.82		USD 667,529.73		USD 1,483,399.39	
**Variable overheads 20%**	USD 277,408.60		USD 416,112.91		USD 924,695.35	
**Total recurrent cost in USD**	**USD 1 , 664 , 451.63**	**0.44**	**USD 2 , 496 , 677.44**	**0.66**	**USD 5 , 548 , 172.09**	**1.48**
**Scenario 3: Utilization 2.00**						
**Consultation costs**	USD 878,669.53		USD 1,318,004.30		USD 2,928,898.44	
**Drug costs**	USD 2,889,423.28		USD 4,334,134.93		USD 9,631,410.95	
**Human resources**	USD 1,780,079.27		USD 2,670,118.90		USD 5,933,597.56	
**Variable overheads 20%**	USD 1,109,634.42		USD 1,664,451.63		USD 3,698,781.39	
**Total recurrent cost in USD**	**USD 6 , 657 , 806.50**	**1.77**	**USD 9 , 986 , 709.76**	**2.66**	**USD 22 , 192 , 688.35**	**5.91**

## Discussion

### Insights into the cost of providing curative outpatient services at first-level healthcare facilities to the poorest and policy implications

Our study estimates the average cost of providing curative outpatient services at first level healthcare facilities to Burkina Faso’s poorest population, ranging from USD 3.17 to USD 5.61 per consultation. These cost estimates offer policymakers valuable insights, particularly when it comes to setting prices for healthcare services covered by free healthcare policies for the poorest supported by the government through the RAMU. Notably, the cost estimate, while specific to curative care, underscores the broader need for accurate financial planning within the healthcare system. It is this accurate financial planning that plays a vital role in ensuring adequate resources for the successful implementation of the free health care for the poor and achieving the global goal of strengthening health systems for UHC, as emphasized in the Declaration of Astana.

Moreover, recognizing the importance of curative outpatient services at first-level facilities as part of primary healthcare (PHC) [33] further emphasizes the significance of our cost estimate. Such services represent the first point of care and are instrumental in the pursuit of comprehensive healthcare for all. Beyond the local context, the involvement of organizations such as the World Bank in discussions about implementing flat-rate purchases for free healthcare policies [34, 35] underlines the practical relevance of our findings and illustrates how these findings can help shape healthcare policy and financial planning in the region.

Our cost estimates align with earlier studies in Burkina Faso for the general population, underscoring a potential stability in healthcare costs even considering the considerable time elapsed since those studies were conducted [29, 36]. Notably, Flessa & Marschall (2009) estimated the average cost per consultation at USD 2.94 [29], while Mugisha et al. (2002) evaluated outpatient services for the rural population in Nouna at USD 3.08 [36], which is almost matching our lowest estimate of USD 3.17. The consistency of these figures over time prompts reflection on how inflation has seemingly had minimal impact on healthcare costs within Burkina Faso’s healthcare system. However, a study in rural Ghana [37] revealed a notably higher median cost of USD 8.79 for outpatient department attendance, likely linked to economic disparities. Ghana’s comparatively higher income level, relative to Burkina Faso, suggests a plausible influence on healthcare expenses.

Drug costs accounted for 35.33% of the total cost, and human resources at 21.77%. Our estimate differs from prior studies, where drug costs were the second-largest driver [36, 37], but it aligns with our initial expectations due to the complex morbidity profile of the poorest population seeking late-stage care requiring complex medication [38]. To enhance access to quality healthcare, controlling drug costs and improving procurement processes are crucial [39]. While not directly derived from our cost analysis, the role of community health workers emerges as a complementary strategy, especially relevant in the context of our findings. By providing first-level services, particularly in underserved rural areas [40], community health workers can alleviate the burden on healthcare facilities, indirectly affecting the cost structure by reducing the demand for more expensive, late-stage treatments. Their involvement can improve medication adherence among the poorest populations, potentially mitigating the need for complex and costly care. This indirect link suggests that incorporating community health workers into healthcare delivery models could enhance the overall cost-effectiveness and efficiency of care for these vulnerable groups.

### Insights into the budget-impact and policy implications

The findings indicate that across all hypothetical budget scenarios (see also Table S1 and Table S2), providing free curative outpatient care at first-level healthcare facilities for up to 20% of the population would result in a healthcare budget impact between 0.22 and 11.45%. This suggests that providing these services to the bottom 20% of the population could be financially viable without imposing a substantial burden on the government’s budget. The baseline scenario estimates costs and impacts based on the current utilization rate of 0.25 healthcare contacts per person per year among the poorest and a community targeting threshold of 6% nationwide. In contrast, the other scenarios explore the budget impact of increased utilization and broader coverage under user fee exemptions. While the baseline scenario provides a foundational estimate, higher utilization and expanded coverage, as illustrated in the alternative scenarios, may offer greater benefits in terms of equity and access to healthcare services. Ultimately, policymakers must select the scenario that best aligns with national budget constraints while advancing towards UHC and ensuring that no one is left behind.

While Burkina Faso allocated 46% of its government health spending to PHC in 2020 [41], a relatively high share compared with neighboring countries (such as Côte d’Ivoire 38%, Niger 36% and Benin 13% [41]) the need for further investment and resource allocation efficiency is evident. This becomes particularly important when considering that 31% of PHC funding in Burkina Faso relies on out-of-pocket payments and 22% on external sources [41]. Policymakers should explore fund reallocation and alternative financing sources [42], bearing in mind the challenges posed by the current economic and security context [12] as they work to progress toward UHC and ensure equitable healthcare access for the most vulnerable populations.

Policymakers may also need to consider implementing RAMU gradually, increasing coverage step by step, as done in other countries with successful fee removal policies, such as Ghana, Colombia, Mexico, and Thailand [43]. Furthermore, our study did not consider the cost of identifying the poorest, estimated at 5.73 USD per beneficiary [44], which can significantly impact the financial costs of the policy and should be factored in to ensure its sustainability and affordability.

With the ongoing implementation of RAMU in Burkina Faso [6], the findings of our study hold significant relevance. Policymakers can use our cost estimates and budget impact analysis to guide budget planning, policy decisions, and the pricing of services. Addressing financial implications, cost drivers, and cost-effectiveness strategies will support the successful and sustainable implementation of RAMU and advance the goals of UHC and LNOB in Burkina Faso and similar resource constrained settings.

Future research could build on our study’s findings by conducting further analyses that combine information on costs with information on health outcomes and financial protection. Preliminary evidence by Atchessi et al. (2016) and Beaugé et al. (2020) have already shown that while targeted user fee exemptions have not lead to significant improvements in healthcare utilization [45, 46], they have resulted in reductions in excessive out-of-pocket expenditures [38].

## Methodological consideration

Our study has limitations that must be considered. Firstly, the cost estimates were derived from a single district in Burkina Faso with a limited number of health facilities, which may limit the generalizability of the findings beyond the country contexts. Secondly, rapid tests and test strips were excluded from the analysis due to their minimal usage within the CSPS facilities studied. While their usage was minimal, this could have potentially led to a slight underestimation of the costs. Thirdly, the treatment mix provided in our study may not fully reflect the quality standard of care offered by facilities to non-poor patients, as practitioners may have focused solely on treating the presented conditions without considering underlying co-morbidities. However, we have triangulated our data with another micro-costing study on health service use among children from the general population, yielding similar findings (unpublished). Moreover, even though providers were compensated for treating the poorest, the incentives provided by the PBF intervention may not have fully encouraged a comprehensive range of services. This discrepancy could have led to a situation where the services provided to the poorest were more limited in scope compared to what was available to other patient groups. To address this limitation, future studies could explore provider behavior through qualitative research.

## Conclusion

Providing curative care services to the poorest at first-level facilities is critical for achieving universal health coverage and leaving no one behind. Our study informs policies such as RAMU in Burkina Faso by providing cost information to plan and finance free curative care for the poor. More research is needed for better cost estimation and the budgeting of curative care services at a higher level of care in LMICs.

## Supplementary Information

Supplementary Material 1.

Supplementary Material 2.

Supplementary Material 3.

Supplementary Material 4.

## Data Availability

The datasets analyzed during the study are available from the corresponding author on request. Data sharing is subject to compliance with ethical guidelines and institutional policies.
