# Supplementary material for: Costing curative outpatient care for the poorest in Burkina Faso: informing universal health coverage and leaving no one behind
Source: BMC Health Serv Res. 2024 Nov 28;24:1497. doi: 10.1186/s12913-024-11854-8 (PMC11603942; doi:10.1186/s12913-024-11854-8)
Supplement: Supplementary file 2 — Supplementary Material 2. [file 12913_2024_11854_MOESM2_ESM.pdf]

QUESTIONNAIRE D'ENQUETE DESTINE AUX FORMATIONS SANITAIRES (CSPS, Structures privées à but non lucratif)

**IDENTIFICATION**
REGION SANITAIRE………………………………………………………………………………………..
DISTRICT:………………………………………………………………………………………………
NOM DE LA FORMATION SANITAIRE:………………………………………………………………… …
COMMUNE :……………………………………………………………………………..
ENQUETEUR:………………………………………………………………………………………….
DATE DE L'ENQUETE:……………………………………………………………………….(JJ.MM.AAAA)

<u>Instructions à L'ENQUETEUR:</u>

- *Au cours de votre séjour d'une semaine prévu dans cette formation sanitaire, veuillez identifier et interroger les personnes clés pour remplir ce questionnaire. Vous pouvez remplir ce questionnaire sur un ou plusieurs jours en fonction de la disponibilité des personnes clés. Vous pouvez pour certaines informations voir le responsable de la formation sanitaire et trianguler l'information recueillie avec le CISSE et le Responsable Administratif et Financier (ex gestionnaire du district).*

Ce questionnaire a pour but de collecter des informations sur la consommation de ressources majeures (intrants qui durent plus d'un an) en relation avec la prise en charge des accouchements et les services de santé pour les enfants de moins de cinq ans dans les 32 formations sanitaires sélectionnées (Centres de Santé et de Promotion Sociale (CSPS), Structures privées à but non lucratif) dans 4 régions du Burkina Faso. Nous évaluerons deux principales catégories de ressources majeures, à savoir le bâtiment et l'équipement. En outre, nous collecterons des informations générales sur les formations sanitaires pour constituer les bases de la répartition des coûts partagés. En conséquence, ce questionnaire se compose de trois sections. La **section 1** recueille les informations générales sur les formations sanitaires enquêtées. La **section 2** recueille les informations sur le bâtiment où se déroule la prise en charge de l'accouchement et les soins ambulatoires de santé pour les enfants de moins de cinq ans (le service de maternité et la salle de consultation). Et la **section 3** recueille les informations sur l'équipement utilisé pour la prise en charge de l'accouchement et les soins de santé pour les enfants de moins de cinq ans.

SECTION 1. Informations générales sur la formation sanitaire enquêtée

Q1. En quelle année cette formation sanitaire a-t-elle été ouverte au public? ☐☐☐☐

(Ici nous voulons l'année d'ouverture au public. Ecrivez un nombre à quatre chiffres pour l'année)

Q2. Où est située cette formation sanitaire?

(Cochez la seule case qui s'applique)

- ☐ Rural
- ☐ Urbain

Q3. Cette formation sanitaire est-elle?

(Cochez la seule case qui s'applique)

- ☐ Public
- ☐ Privé à but non lucratif

Q4. Combien de personnes sont employées dans cette formation sanitaire ?

(Ecrivez le nombre total de tous les membres du personnel de l'Etat (fonctionnaires) et agents de soutien sur la liste de paie ayant un contrat de travail avec cette formation sanitaire, qu'il soit temporaire ou permanent. Inscrivez le nombre de personnes. Inscrivez 01, 02, 03 pour les nombres inférieurs à 10. Ne pas inclure les agents de santé à base communautaire).

Nombre total de personnes employées ☐☐

Q5. Parmi les personnes employées ci-dessus, combien d'agents de santé participent au suivi de la femme en travail, de l'accouchement et de la surveillance du post-partum dans cette formation sanitaire?

(Ecrivez le nombre total de tous les membres du personnel inscrits dans le registre du personnel avec un contrat de travail avec cette formation sanitaire, qu'il soit temporaire ou permanent et qui participent au suivi de la femme en travail, de l'accouchement et de la surveillance. Inscrivez le nombre de personnes Inscrivez 01, 02, 03 pour les nombres inférieurs à 10).

Nombre total des agents de santé participant au suivi de la femme en travail, de l'accouchement et de la surveillance du post-partum: ☐☐

Q6. Veuillez indiquer les agents de santé participant au suivi de la femme en travail, de l'accouchement et de la surveillance du post-partum ci-dessus mentionnés.

(Inscrivez l'agent de santé participant au suivi de la femme en travail, de l'accouchement et de la surveillance du post-partum, en utilisant le code approprié fourni ci-dessous. Utilisez chaque ligne pour chaque agent de santé. Veuillez ajouter plusieurs lignes au besoin)

**Code Agent de santé**	**Qualification du personnel**	**Nombre**
1	Infirmier Diplômé d'Etat	
2	Sage-femme ou maïeuticien d'Etat	
3	Infirmier Breveté	
4	Accoucheuse brevetée	
5	Agent Itinérant de Santé	
6	Fille de salle/ Garçon de salle	
7	Brancardier	
8	Autre, précisez	
9		
10		

Q7. Parmi les personnes employées, combien d'agents de santé participent à la consultation des enfants de moins de cinq ans dans cette formation sanitaire?

(Ecrivez le nombre total de tous les membres du personnel de l'Etat (fonctionnaires) et agents de soutien sur la liste de paie ayant un contrat de travail avec cette formation sanitaire, qu'il soit temporaire ou permanent. Inscrivez le nombre de personnes. Inscrivez 01, 02, 03 pour les nombres inférieurs à 10. Ne pas inclure les agents de santé à base communautaire).

Nombre total des agents de santé participant à la consultation des enfants de moins de cinq ans: ☐☐

Q8. Veuillez indiquer les agents de santé participant à la consultation des enfants de moins de cinq ans ci-dessus mentionnés.

(Inscrivez l'agent de santé **participant à la consultation des enfants de moins de cinq ans***, en utilisant le code approprié fourni ci-dessous. Utilisez chaque ligne pour chaque agent de santé. Veuillez ajouter plusieurs lignes au besoin)*

**Code Agent de santé**	**Qualification du personnel**	**Nombre**
1	Infirmier Diplômé d'Etat	
2	Sage-femme ou maïeuticien d'Etat	
3	Infirmier Breveté	
4	Accoucheuse brevetée	
5	Agent Itinérant de Santé	
6	Autre, précisez	
7		
8		

Q9. Combien d'accouchements ont été réalisés mensuellement dans cette formation sanitaire entre Janvier 2017 et décembre 2017?

(Inscrivez le nombre total des accouchements réalisés y compris les accouchements compliqués

Janv. 2017	Fevr. 2017	Mars. 2017	Avr. 2017	Mai 2017	Juin. 2017	Juil. 2017	Aout. 2017	Sept. 2017	Oct. 2017	Nov. 2017	Dec. 2017

Q10. Combien de femmes en travail ont été évacuées à l'échelon supérieur mensuellement entre Janvier 2017 et décembre 2017?

(Inscrivez le nombre total de femmes en travail évacuées à l'échelon supérieur)

Janv. 2017	Fevr. 2017	Mars. 2017	Avr. 2017	Mai 2017	Juin. 2017	Juil. 2017	Aout. 2017	Sept. 2017	Oct. 2017	Nov. 2017	Dec. 2017

Q11. Combien de femmes accouchées récentes ont été évacuées à l'échelon supérieur mensuellement entre janvier 2017 et décembre 2017?

(Inscrivez le nombre total de femmes accouchées récentes évacuées à l'échelon supérieur)

Janv. 2017	Fevr. 2017	Mars. 2017	Avr. 2017	Mai 2017	Juin. 2017	Juil. 2017	Aout. 2017	Sept. 2017	Oct. 2017	Nov. 2017	Dec. 2017

Q12. Combien d'enfants de moins de cinq (y compris les nouveau-nés) ont été évacués à l'échelon supérieur mensuellement entre Janvier 2017 et décembre 2017?

(Inscrivez le nombre total d'enfants de moins de cinq y compris les nouveau-nés évacué à l'échelon supérieur. Comptabiliser les moins de cinq ans de la maternité et de la pédiatrie.)

Janv. 2017	Fevr. 2017	Mars. 2017	Avr. 2017	Mai 2017	Juin. 2017	Juil. 2017	Aout. 2017	Sept. 2017	Oct. 2017	Nov. 2017	Dec. 2017

Q13. Combien de consultations curatives externes ont été effectuées mensuellement dans cette formation sanitaire entre janvier 2017 et décembre 2017??

(Veuillez noter le nombre de consultations curatives externes effectuées mensuellement dans cette formation sanitaire, y compris les consultations des adultes et des enfants de tous âges, entre janvier 2017 et décembre 2017)

Janv. 2017	Fevr. 2017	Mars. 2017	Avr. 2017	Mai 2017	Juin. 2017	Juil. 2017	Aout. 2017	Sept. 2017	Oct. 2017	Nov. 2017	Dec. 2017

Q14. Combien de consultations d'enfants de moins de cinq ans ont été effectués mensuellement dans cette formation sanitaire entre janvier 2017 et décembre 2017?

(Inscrivez le nombre total de consultation réalisés)

Janv. 2017	Fevr. 2017	Mars. 2017	Avr. 2017	Mai 2017	Juin. 2017	Juil. 2017	Aout. 2017	Sept. 2017	Oct. 2017	Nov. 2017	Dec. 2017

Q.15 Veuillez indiquer les dépenses relatives aux frais généraux dans cette formation sanitaire, de janvier 2017 à décembre 2017.

(En cas de discordances dans les données, privilégier celles fournies par le responsable du CSPS. Veuillez entrer le total des dépenses pour la période de 12 mois allant de janvier 2017 à décembre 2017 pour chaque élément listé dans le tableau ci-dessous. Veuillez écrire 99 pour les éléments pour lesquels aucune donnée n'est disponible dans la formation sanitaire enquêtée. Veuillez également préciser si c'est la mairie qui fournit les différents éléments.)

No.	Catégories de frais généraux	Montant
1	Electricité	
2	Eau	
3	Téléphone mobile	
4	Entretien des locaux (nettoyage)	
5	Produits d'entretiens des instruments (eau chlorée, formol etc.)	
6	Réparations et maintenance (parc automobile y compris les motos)	
7	Réparations et maintenance (appareils)	
8	Gaz	
9	Pétrole	
10	Carburant	
10	Achats de registres	
11	Fournitures de bureau	
12	Ordonnanciers/bulletins d'examens	
13	Utilisation Plaque solaire (si possible précisez le prix et l'année d'installation)	
14	Autre, précisez	
15		
16		
17		

Q16. Veuillez faire une copie de la tarification des actes, des services, des médicaments et tests fournis dans cette formation sanitaire et attacher cette copie à la fin de ce questionnaire (Voir le responsable du CSPS) *(Lorsque vous faites une copie, assurez-vous que le nom de la formation sanitaire est clairement indiqué sur la copie. Quand ce n'est pas possible de faire une copie, vous pouvez soit écrire vous-même toutes les informations sur la tarification des actes, des services, des médicaments et des tests sur une feuille blanche, soit faire une photo de la tarification avec votre smartphone si vous en avez un. Dans les deux situations, vous devrez vous assurer que le nom de la formation sanitaire est clairement indiqué.)*

---Fin de la section 1---

SECTION 2. Informations sur la construction du service de maternité et celui de soins ambulatoires (consultations externes) de cette formation sanitaire

2.1. Informations sur la construction du service de maternité de cette formation sanitaire

Q1. En quelle année a été construit le bâtiment de la maternité de cette formation sanitaire?

(Ici nous voulons l'année d'ouverture au public. Ecrivez un nombre à quatre chiffres pour l'année)

Année de construction: ☐☐☐☐

Q2. Combien de pièces y a-t-il dans la maternité de cette formation sanitaire?

(Inscrivez le nombre de pièces Inscrivez 01, 02, 03 pour les nombres inférieurs à 10)

Nombre de pièces dans la maternité: ☐☐

Q3. Combien de bâtiments y a-t-il dans le service de maternité de cette formation sanitaire? *(Veuillez compter et noter le nombre total de bâtiments qui appartiennent au service de maternité de cette formation sanitaire. Ecrivez 01, 02, 03 etc. si c'est en-dessous de 10)*

Nombre de bâtiments dans le service de maternité : ☐☐

Q4. Veuillez compter le nombre d'étages et effectuer la mesure physique du rez-de-chaussée de tous les bâtiments que vous avez comptés à Q3 ci-dessus. Indiquez alors le nombre d'étages et la surface du rez-de-chaussée de chaque bâtiment dans le tableau ci-dessous *(Veuillez effectuer la mesure physique du rez-de-chaussée de chaque bâtiment comptabilisé en Q3. La mesure doit être effectuée à l'extérieur des bâtiments et la zone mesurée doit inclure les parties communes (par exemple les couloirs, les escaliers). Vous pouvez ne pas avoir besoin d'effectuer les mesures si vous pouvez vous procurer le plan du service de maternité avec les différentes dimensions. Veuillez alors en faire une copie, une photo ou renseigner directement le tableau ci-dessous à partir de ce plan. Ecrivez 01, 02, 03 etc. pour les chiffres inférieurs à 10.)*

**Numéro de bâtiment**	**Nombre d'étages**	**Mesures du rez-de-chaussée**	
		**Largeur (m)**	**Longueur (m)**
Bâtiment 1			
Bâtiment 2			
Bâtiment 3			

…..			

<u>2.2. Informations sur la construction du service de soins ambulatoires (consultations externes) de cette formation sanitaire</u>

Q1. En quelle année a été construit le <u>service de soins ambulatoires (consultations externes)</u> de cette formation sanitaire?

(Ici nous voulons l'année d'ouverture au public. Ecrivez un nombre à quatre chiffres pour l'année)

Année de construction: ☐☐☐☐

Q2. Combien de pièces sont utilisées pour la consultation des enfants de moins de cinq ans?

(Inscrivez le nombre de pièces. Inscrivez 01, 02, 03 pour les nombres inférieurs à 10)

Nombre de pièces : ☐☐

Q3. Combien de bâtiments y a-t-il dans le service de soins ambulatoires (consultations externes) de cette formation sanitaire? *(Veuillez compter et noter le nombre total de bâtiments qui appartiennent au service de soins ambulatoires (consultations externes) de cette formation sanitaire. Ecrivez 01, 02, 03 etc. si c'est en-dessous de 10)*

Nombre de bâtiments dans le service de soins ambulatoires (consultations externes) :

☐☐

Q4. Veuillez compter le nombre d'étages et effectuer la mesure physique du rez-de-chaussée de tous les bâtiments que vous avez comptés à Q3 ci-dessus. Indiquez alors le nombre d'étages et la surface du rez-de-chaussée de chaque bâtiment dans le tableau ci-dessous *(Veuillez effectuer la mesure physique du rez-de-chaussée de chaque bâtiment comptabilisé en Q3. La mesure doit être effectuée à l'extérieur des bâtiments et la zone mesurée doit inclure les parties communes (par exemple les couloirs, les escaliers). Vous pouvez ne pas avoir besoin d'effectuer les mesures si vous pouvez vous procurer le plan du service de maternité avec les différentes dimensions. Veuillez alors en faire une copie, une photo ou renseigner directement le tableau ci-dessous à partir de ce plan. Ecrivez 01, 02, 03 etc. pour les chiffres inférieurs à 10.)*

Numéro de bâtiment	Nombre d'étages	Mesures du rez-de-chaussée	
		**Largeur (m)**	**Longueur (m)**
Bâtiment 1			
Bâtiment 2			
Bâtiment 3			
…..			

---Fin de la Section 2---

SECTION 3. Informations sur l'équipement utilisé dans le service de maternité, de la salle de consultation (ou salle PCIME si applicable) et des bureaux

Q.1 Veuillez indiquer la quantité d'équipement qui se trouve dans la maternité de cette formation sanitaire dans le tableau ci-dessous. Veuillez indiquer également où se trouve cet équipement? *(Remplir le tableau avec l'aide des agents de santé si nécessaire en comptant et inscrivant le nombre d'articles disponibles. Indiquer la pièce où se trouve l'équipement en utilisant les codes de pièce appropriés fournis. Ne pas comptabiliser le matériel neuf stocké au magasin)*

No.	Nom et catégorie de l'équipement	Quantité utilisée	**Emplacement de l'équipement**<br>*1 = Salle des consultations prénatales*<br>*2 = salle d'accouchement*<br>*3 = Salle du post-partum*<br>*4 = Salle de stérilisation*<br>*5 = Salles pour d'autres activités*
1	Table d'examen gynécologique avec étriers		
2	Autres tables d'examen gynécologique (précisez)		

3	Table d’accouchement		
4	Bassin de lit		
5	Potence		
6	Lit		
7	Matelas		
8	Ventilateurs sur pieds		
9	Ventilateurs au plafond/Muraux		
10	Escabeau		
11	Table consultation (bureau)		
12	Chaises		
13	Bancs		
14	Armoires		
15	Pèse personne		
16	Toise		
17	Pèse personne combinée à la toise		
18	Horloge pour la salle d’accouchement		
19	Tensiomètre adulte (préciser si à mercure, sphyngomanomètre ou électronique)		
20	Stéthoscope médical		
21	Stéthoscope obstétrical		
22	Thermomètre médical (précisez si à mercure, laser, électronique)		
23	Mètre ruban		
24	Spéculum		
25	Source lumineuse électrique (sur pieds)		
26	Source lumineuse (lampe-torche)		
27	Tambour à compresses		

28	Boîte d'accouchement		
29	Boîte de suture d'épisiotomie		
30	Boîte d'instruments gynécologiques (pinces,…)		
31	Chariot de soins/ Table servant de soins		
32	Haricot/ plateau de soins		
33	Tambour à coton		
34	Bac de décontamination		
35	Matériel de garde d'eau (seau, barrique, jarre etc.)		
36	Autoclave		
37	Poupinel		
38	Réchaud à gaz		
39	Bouteille de gaz butane 12 Kg		
40	Bouteille de gaz butane 6 Kg		
41	Détendeur + raccord de gaz butane		
42	Table de soins habituels du nouveau-né		
43	Table de réanimation du nouveau-né		
44	Matériel d'aspiration du nouveau-né (pingouin, poire)		
45	Aspirateur électrique de mucosités		
46	Kit de réanimation complet		
47	Horloge pour la salle de réanimation		
48	Pèse bébé		
49	Stéthoscope médical pour réanimation du nouveau-né		
50	Ventouse		
51	Tambour à champs		

52	Autre, précisez		
53			
54			
55			
56			

Q.2 Veuillez indiquer la quantité d'équipement qui se trouve dans la salle de consultation (ou salle PCIME si applicable) dans le tableau ci-dessous? Veuillez indiquer également où se trouve cet équipement? *(Remplir le tableau avec l'aide des agents de santé si nécessaire en comptant et inscrivant le nombre d'articles disponibles. Indiquer la pièce où se trouve l'équipement en utilisant les codes de pièce appropriés fournis. Ne pas comptabiliser le matériel neuf stocké au magasin.)*

No.	Nom et catégorie de l'équipement	Quantité utilisée	**Emplacement de l'équipement**<br>*1 = Salle de consultation*<br>*2 = salle de mise en observation*<br>*3 = hall d'attente*<br>*4= Salle de réunion*<br>*5 = Salle de pansement*<br>*6 = Salle de Soins et de petite chirugie*<br>*7= Salles pour d'autres activités*
1	Table d'examen		
2	Banc d'attente		

3	Tableau d'affichage		
4	Bureau à 1 caisson		
5	Chaise de bureau		
6	Chaise visiteur		
7	Armoire à dossiers murale		
8	Armoire à 2 battants		
9	Horloge murale		
10	Tonnelet avec robinet 50 litres		
11	Escabeau à 2 marches		
12	Otoscope à piles		
13	Pèse bébé		
14	Pèse personne avec toise		
15	Stéthoscope médical		
16	Tensiomètre enfant		
17	Tensiomètre adulte (précisez si à mercure, sphyngomanomètre ou électronique)		
18	Poubelle à pédale		
19	Thermomètre médical		
20	Bocal porte instruments		
21	Boîte à coton		
22	Haricot 26 cm		
23	Pince de Péan droite 14 cm		
24	Plateau inox rectangulaire moyen		
25	Plateau inox rectangulaire grand		
26	Plateau inox rectangulaire petit		
27	Potence à perfusion en acier inoxydable, double crochets		
28	Mètre ruban		
29	Source lumineuse (lampe-torche)		

30	Tambour à compresses		
31	Tabouret praticien		
32	Table consultation (bureau)		
33	Pince de Kocher		
34	Paravent		
35	Pissette à alcool plastique		
36	Tambour/boite à coton		
37	Bassin pour décontamination		
38	Matériel de garde d'eau (seau, barrique, jarre etc.)		
39	Autoclave type cocotte		
40	Poupinel		
41	Réchaud à gaz		
42	Bouteille de gaz butane 12 Kg		
43	Bouteille de gaz butane 6 Kg		
44	Détendeur + raccord de gaz butane		
45	Chariot de soins		
46	Pince à servir		
47	Pingouin, poire		
48	Bassin de soins		
49	Boîte de petite chirurgie		
50	Lampe d'examen		
51	Table de pansement		
52	Boîte à gants		
53	Glacière porte vaccin		

54	Réfrigérateur mixte (gaz/ électricité)		
55	Accumulateur de froid (Ice box)		
56	Autre, précisez		
57			
58			
59			

Q3. S'il vous plaît, veuillez indiquer la quantité de matériel de bureau qui se trouve au service de maternité et de consultations externes (salle PCIME si applicable) dans cette formation sanitaire. Veuillez indiquer également où est ce qu'il est situé? *(Remplir le tableau avec l'aide des agents de santé si nécessaire en comptant et inscrivant le nombre d'articles disponibles. Indiquer la pièce où se trouve l'équipement en utilisant les codes de pièce appropriés fournis. Ne pas comptabiliser le matériel neuf stocké au magasin)*

**No.**	**Catégorie et nom de l'équipement**	**Quantité utilisée**	**Localisation de l'équipement**<br>*1 = Service de maternité*<br>*2 = Salle de consultation (salle PCIME si applicable)*<br>*3 = Salle pour d'autres activités*
1	Ordinateur portable		
2	Ordinateur de bureau		
3	Télephone fixe		
4	Fax		
5	Photocopieuse		
6	Imprimante		
7	Climatiseur		
8	Groupe électrogène		
9	Autre, précisez		

10			
11			
12			
13			
