# Supplementary material for: Costing curative outpatient care for the poorest in Burkina Faso: informing universal health coverage and leaving no one behind
Source: BMC Health Serv Res. 2024 Nov 28;24:1497. doi: 10.1186/s12913-024-11854-8 (PMC11603942; doi:10.1186/s12913-024-11854-8)
Supplement: Supplementary file 3 — Supplementary Material 3. [file 12913_2024_11854_MOESM3_ESM.pdf]

QUESTIONNAIRE SURVEY AT AT TRAINING SANITARY (CSPS, private non-profit structures)

**IDENTIFICATION**
HEALTH REGION..
DISTRICT:.. HEALTH FACILITY
NAME:... ...
COMMUNE:..
INVESTIGATOR:... DATE OF SURVEY
....(DD.MM.YYYY)

Instructions to THE INVESTIGATOR:

- *During your planned one-week stay in this health facility, please identify and interview key people to complete this questionnaire. You can complete this questionnaire over one or more days, depending on the availability of key people. For certain information, you can see the health facility manager and triangulate the information gathered with the CISSE and the Administrative and Financial Manager (ex district manager).*

The aim of this questionnaire is to collect information on the consumption of major resources (inputs that last more than one year) in relation to the management of childbirth and health services for children under five in 32 selected health facilities (Centres de Santé et de Promotion Sociale (CSPS), private non-profit structures) in 4 regions of Burkina Faso. We will evaluate two main categories of major resources, namely buildings and equipment. In addition, we will collect general information on the health facilities to form the basis for the allocation of shared costs. Accordingly, this questionnaire is divided into three sections. **Section 1** collects general information on the health facilities surveyed. **Section 2** collects information on the building where childbirth and ambulatory health care for children under five is provided (the maternity ward and the consultation room). And **section 3** collects information on the equipment used for the management of childbirth and health care for children under five.

SECTION 1. General information on the health facility surveyed

Q1. In what year was this health facility opened to the public? ☐☐☐☐

(Here we want the year of opening to the public. Write a four-digit number for the year)

Q2. Where is this health facility located?

(Check only the box that applies)

☐ Rural☐
Urban

Q3. Is this health facility?

(Check only the box that applies)

☐ Public
☐ Private non-profit

Q4. How many people are employed in this health facility?

(Write the total number of all government employees (civil servants) and support staff on the payroll who have an employment contract with this health facility, whether temporary or permanent. Enter the number of people. Enter 01, 02, 03 for numbers less than 10 (do not include community health workers).

Total number of employees

☐☐

Q5. Of the above, how many health workers are involved in monitoring women in labour, delivery and post-partum care in this health facility?

(Write the total number of all staff listed in the staff register with an employment contract with this health facility, whether temporary or permanent, and who are involved in monitoring women in labor, delivery and surveillance. Enter the number of persons (01, 02, 03 for numbers less than 10).

Total number of health workers involved in monitoring women in labor, delivery and post-partum care: ☐☐

Q6. please indicate the health workers involved in the above-mentioned monitoring of women in labour, delivery and post-partum care.

(Enter the health worker involved in monitoring the woman in labor, delivery and postpartum surveillance, using the appropriate code provided below. Use each line for each health worker. Please add more than one line if necessary)

Code Health agent	Staff qualifications	Number
1	State-qualified nurse	
2	State-qualified midwife or maieutician	
3	Registered Nurse	
4	Patented midwife	
5	Itinerant Health Agent	
6	Hall girl/ Hall boy	
7	Stretcher-bearer	
8	Other, please specify	
9		
10		

Q7. Of the people employed, how many health workers are involved in consultations with children under five in this health facility?

(Write the total number of all government employees (civil servants) and support staff on the payroll who have an employment contract with this health facility, whether temporary or permanent. Enter the number of people. Enter 01, 02, 03 for numbers less than 10 (do not include community health workers).

Total number of health workers involved in consultations with children under five:

☐☐

Q8. Please indicate the health workers involved in the consultation of the above-mentioned children under the age of five.

(Enter the health worker **involved in the consultation for children under five***, using the appropriate code provided below. Use each line for each health worker. Please add more than one line if necessary)*

Code Health agent	Staff qualifications	Number
1	State-qualified nurse	
2	State-qualified midwife or maieutician	
3	Registered Nurse	
4	Patented midwife	
5	Itinerant Health Agent	
6	Other, please specify	
7		
8		

Q9. How many deliveries were carried out monthly in this health facility between January 2017 and December 2017?

(Enter the total number of births performed, including complicated deliveries.

Jan. 2017	Feb. 2017	Mars. 2017	Apr. 2017	May 2017	June. 2017	Jul. 2017	August. 2017	Sept. 2017	Oct. 2017	Nov. 2017	Dec. 2017

Q10. How many working women were evacuated to the next level monthly between January 2017 and December 2017?

(Enter the total number of working women evacuated to the next level)

Jan. 2017	Feb. 2017	Mars. 2017	Apr. 2017	May 2017	June. 2017	Jul. 2017	August. 2017	Sept. 2017	Oct. 2017	Nov. 2017	Dec. 2017

Q11. How many recently delivered women were evacuated to the upper echelon monthly between January 2017 and December 2017?

(Enter the total number of women who have recently given birth and have been evacuated to the next higher level)

Jan. 2017	Feb. 2017	Mars. 2017	Apr. 2017	May 2017	June. 2017	Jul. 2017	August. 2017	Sept. 2017	Oct. 2017	Nov. 2017	Dec. 2017

Q12. How many children under five (including newborns) were evacuated to the next level monthly between January 2017 and December 2017?

(Enter the total number of children under five, including newborns, evacuated to the next level. Count under-fives in maternity and pediatrics).

Jan. 2017	Feb. 2017	Mars. 2017	Apr. 2017	May 2017	June. 2017	Jul. 2017	August. 2017	Sept. 2017	Oct. 2017	Nov. 2017	Dec. 2017

Q13. How many curative outpatient consultations were carried out monthly in this health facility between January 2017 and December 2017?

(Please note the number of curative outpatient consultations performed monthly in this health facility, including consultations for adults and children of all ages, between January 2017 and December 2017)

Jan. 2017	Feb. 2017	Mars. 2017	Apr. 2017	May 2017	June. 2017	Jul. 2017	August. 2017	Sept. 2017	Oct. 2017	Nov. 2017	Dec. 2017

Q14. How many consultations for children under five were carried out monthly in this health facility between January 2017 and December 2017? *(Write down the total number of consultations carried out)*

Jan. 2017	Feb. 2017	Mars. 2017	Apr. 2017	May 2017	June. 2017	Jul. 2017	August. 2017	Sept. 2017	Oct. 2017	Nov. 2017	Dec. 2017

Q.15 Please indicate the expenditure on overheads in this health facility, from January 2017 to December 2017.

(In the event of discrepancies in the data, prefer those provided by the CSPS manager. Please enter the total expenditure for the 12-month period from January 2017 to December 2017 for each item listed in the table below. Please write 99 for items for which no data is available in the health facility surveyed. Please also specify whether the mayor's office provides the various items).

No.	Overhead categories	Amount
1	Electricity	
2	Water	
3	Cell phone	
4	Maintenance of premises (cleaning)	
5	Instrument care products (chlorinated water, formaldehyde etc.)	
6	Repairs and maintenance (vehicle fleet y including motorcycles)	
7	Repairs and maintenance (equipment)	
8	Gas	
9	Oil	
10	Fuel	
10	Register purchases	
11	Office supplies	
12	Prescription pads/examination forms	
13	Use of solar panel (if possible, specify type) price and year of installation)	
14	Other, please specify	
15		
16		
17		

Q16. Please make a copy of the fees for procedures, services, medicines and tests provided in this health facility and attach this copy to the end of this questionnaire (See CSPS manager) *(When making a copy, please ensure that the name of the health facility is clearly indicated on the copy. When it's not possible to make a copy, you can either write all the information on the pricing of procedures, services, medicines and tests yourself on a blank sheet of paper, or take a photo of the pricing with your smartphone if you have one. In both cases, you'll need to make sure that the name of the health facility is clearly indicated).*

---End of section 1—

SECTION 2. Information on the construction of the maternity ward and the outpatient department of this health facility

2.1. Information on the construction of the maternity ward at this health facility Q1. In what year was the maternity unit built?

(Here we want the year of opening to the public. Write a four-digit number for the year)

Year of construction: □□□□

Q2. How many rooms are there in the maternity ward of this health facility?

(Enter the number of pieces Enter 01, 02, 03 for numbers less than 10)

Number of rooms in the maternity ward: □□

Q3. How many buildings are there in this f a c i l i t y 's maternity ward? *(Please count and record the total number of buildings that belong to this facility's maternity ward. Write 01, 02, 03 etc. if less than 10)*

Number of buildings in the maternity ward : □□

Q4. Please count the number of floors and physically measure the ground floor of all the buildings you counted in Q3 above. Then enter the number of storeys and the first floor area of each building in the table below *(Please carry out the physical measurement of the first floor of each building counted in Q3. The measurement must be taken outside the buildings, and the area measured must include common areas (e.g. corridors, staircases). You may not need to take measurements if you can obtain a plan of the maternity ward with the various dimensions. Please make a copy, take a photo or fill in the table below directly from the plan. Write 01, 02, 03 etc. for numbers under 10).*

**Number n umber**	**Number of floors**	**First floor measurements**	
		**Width (m)**	**Length (m)**
Building 1			
Building 2			
Building 3			
.....			

2.2. Information on the construction of the outpatient department at this health facility

Q1. In what year was the outpatient department of this health facility built?

(Here we want the year of opening to the public. Write a four-digit number for the year)

Year of construction: ☐☐☐☐

Q2. How many rooms are used for consultations with children under five?

(Enter the number of pieces. Enter 01, 02, 03 for numbers less than 10)

Number of pieces : ☐☐

Q3. How many buildings are there in this facility's outpatient department? *(Please count and record the total number of buildings that belong to this facility's outpatient department. Write 01, 02, 03 etc. if less than 10)*

Number of buildings in the outpatient department :

☐☐

Q4. Please count the number of floors and physically measure the ground floor of all the buildings you counted in Q3 above. Then enter the number of storeys and the first floor area of each building in the table below *(Please carry out the physical measurement of the first floor of each building counted in Q3. The measurement must be taken outside the buildings, and the area measured must include common areas (e.g. corridors, staircases). You may not need to take measurements if you can obtain a plan of the maternity ward with the various dimensions. Please make a copy, take a photo or fill in the table below directly from the plan. Write 01, 02, 03 etc. for numbers under 10).*

Number<br>n<br>umber	Number of floors	First floor measurements	
		Width (m)	Length (m)
Building 1			
Building 2			
Building 3			
.....			

---End of Section 2—

SECTION 3. Information on equipment used in the maternity ward, consultation room (or IMCI room if applicable) and offices

Q.1 Please indicate the quantity of equipment in the maternity ward of this health facility in the table below. Please also indicate where this equipment is located. *(Complete the table with the help of health workers if necessary, counting and recording the number of items available. Indicate the room where the equipment is located, using the appropriate part codes provided. Do not count new equipment stored in the warehouse)*

No.	Equipment name and category	Quantity used	**Location of equipment**<br>*1 = Room of prenatal consultations*<br>*2 = delivery room*<br>*3 = Postpartum room*<br>*4 = Sterilization room*<br>*5 = Rooms for other activities*
1	Gynecological examination table with stirrups		
2	Other gynecological examination tables (specify)		

3	Delivery table		
4	Bed pan		
5	Stem		
6	Bed		
7	Mattresses		
8	Free-standing fans		
9	Ceiling/wall fans		
10	Stepladder		
11	Consultation table (desk)		
12	Chairs		
13	Benches		
14	Cabinets		
15	Bathroom scales		
16	Height gauge		
17	Personal scale combined with height gauge		
18	Delivery room clock		
19	Adult sphygmomanometer (specify mercury, sphyngomanometer or electronic)		
20	Medical stethoscope		
21	Obstetrical stethoscope		
22	Clinical thermometer (specify mercury, laser, electronic)		
23	Tape measure		
24	Speculum		
25	Source light electric (free-standing)		
26	Light source (flashlight)		
27	Compress drum		

28	Delivery box		
29	Episiotomy suture box		
30	Box instruments gynaecological instruments (forceps, etc.)		
31	Care trolley/ Care table		
32	Bean/ care tray		
33	Cotton drum		
34	Decontamination tank		
35	Hardware fromguard equipment (bucket, barrel, jar etc.)		
36	Autoclave		
37	Poupinel		
38	Gas stove		
39	Butane gas bottle 12 Kg		
40	Butane gas cylinder 6 Kg		
41	Regulator + butane gas connector		
42	Routine newborn care table		
43	Newborn resuscitation table		
44	Newborn suction equipment (penguin, bulb)		
45	Electric suction unit		
46	Complete resuscitation kit		
47	Resuscitation room clock		
48	Baby scales		
49	Medical stethoscope for neonatal resuscitation		
50	Suction cup		
51	Field drum		

52	Other, please specify		
53			
54			
55			
56			

Q.2 Please indicate the quantity of equipment in the consultation room (or IMCI room if applicable) in the table below? Please also indicate where this equipment is located. *(Complete the table with the help of health workers if necessary, counting and recording the number of items available. Indicate the room where the equipment is located, using the appropriate part codes provided. Do not count new equipment in store).*

No.	Equipment name and category	Quantity used	Location of equipment<br>*1 = Consultation room*<br>*2 = observation room*<br>*3 = waiting hall*<br>*4= Meeting room*<br>*5 = Dressing room*<br>*6 = Treatment and minor surgery room*<br>*7= Rooms for other activities*
1	Examination table		
2	Waiting bench		

3	Scoreboard		
4	1 pedestal desk		
5	Office chair		
6	Visitor chair		
7	Wall-mounted file cabinet		
8	2-leaf cabinet		
9	Wall clock		
10	Barrel with tap 50 liters		
11	2-step stepladder		
12	Battery-operated otoscope		
13	Baby scales		
14	Bathroom scale with toise		
15	Medical stethoscope		
16	Children's sphygmomanometer		
17	Adult sphygmomanometer (specify mercury, sphyngomanometer or electronic)		
18	Pedal garbage can		
19	Clinical thermometer		
20	Instrument jar		
21	Cotton box		
22	Bean 26 cm		
23	Straight Pean clamp 14 cm		
24	Medium rectangular stainless steel tray		
25	Large rectangular stainless steel tray		
26	Rectangular stainless steel tray small		
27	Stainless steel infusion pole, double hooks		
28	Tape measure		
29	Light source (flashlight)		

30	Compress drum		
31	Practitioner's stool		
32	Consultation table (desk)		
33	Kocher pliers		
34	Screen		
35	Plastic alcohol flask		
36	Cotton drum/box		
37	Decontamination basin		
38	Water storage equipment (buckets, barrels, jars, etc.)		
39	Autoclave cocotte type		
40	Poupinel		
41	Gas stove		
42	Butane gas bottle 12 Kg		
43	Butane gas cylinder 6 Kg		
44	Regulator + butane gas connector		
45	Care cart		
46	Serving tongs		
47	Penguin, pear		
48	Care basin		
49	Small surgery box		
50	Examination lamp		
51	Dressing table		
52	Glove box		
53	Vaccine cooler		

54	Combined refrigerator (gas/electricity)		
55	Cold accumulator (Ice box)		
56	Other, please specify		
57			
58			
59			

Q3. Please indicate the quantity of office equipment located in the maternity and outpatient department (IMCI room if applicable) in this health facility. Please also indicate where it is located. *(Complete the table with the help of health workers if necessary, counting and recording the number of items available. Indicate the room where the equipment is located, using the appropriate part codes provided. Do not count new equipment stored in the warehouse)*

No.	Equipment category and name	Quantity used	**Location of equipment**<br>*1 = Maternity ward*<br>*2 = Consultation room (IMCI room if applicable)*<br>*3 = Room for other activities*
1	Laptop computer		
2	Desktop computer		
3	Fixed-line telephone		
4	Fax		
5	Photocopier		
6	Printer		
7	Air conditioner		
8	Generator		
9	Other, please specify		

10			
11			
12			
13			
