# Supplementary material for: Costing curative outpatient care for the poorest in Burkina Faso: informing universal health coverage and leaving no one behind
Source: BMC Health Serv Res. 2024 Nov 28;24:1497. doi: 10.1186/s12913-024-11854-8 (PMC11603942; doi:10.1186/s12913-024-11854-8)
Supplement: Supplementary file 4 — Supplementary Material 4. [file 12913_2024_11854_MOESM4_ESM.docx]

**Supplementary files**

**Table S1** presents the results from scenario analysis I, applying a recurrent cost of USD 3.84 per consultation. The annual costs would be USD 1,240,278.26, equivalent to 0.33 % of Burkina Faso's healthcare budget in 2019, assuming a target population of 6% and 0.25 curative contacts. Expanding the target population to 20% and the curative contacts to 0.50 would result in a cost of USD 8,268,521.73, equivalent to 2.20 % of Burkina Faso's healthcare budget in 2019.

**Table S1.** Scenario analysis I: Cost and budget impact estimates applying different targeting thresholds and population coverage.

| **Cost category** | **Base case: Targeting threshold 6% of the population:1,290,611** | **% of the healthcare budget** | **Medium assumption scenario: Targeting threshold 9% of the population: 1,935,916** | **% of the healthcare budget** | **High assumption scenario Targeting threshold 20% of the population: 4,302,036** | **% of the healthcare budget** |
| --- | --- | --- | --- | --- | --- | --- |
| **Utilization 0.25** |  |  |  |  |  |  |
| Consultation costs | USD 109,833.69 |  | USD 164,750.54 |  | USD 366,112.31 |  |
| Drug costs | USD 659,879.01 |  | USD 989,818.51 |  | USD 2,199,596.69 |  |
| Human resources | USD 222,509.91 |  | USD 333,764.86 |  | USD 741,699.70 |  |
| Variable overheads 25% | USD 248,055.65 |  | USD 372,083.48 |  | USD 826,852.17 |  |
| **TOTAL RECURRENT COST IN USD** | **USD 1,240,278.26** | **0.33** | **USD 1,860,417.39** | **0.50** | **USD 4,134,260.87** | **1.10** |
| **Utilization 0.50** |  |  |  |  |  |  |
| Consultation costs | USD 219,667.38 |  | USD 329,501.07 |  | USD 732,224.61 |  |
| Drug costs | USD 1,319,758.02 |  | USD 1,979,637.02 |  | USD 4,399,193.39 |  |
| Human resources | USD 445,019.82 |  | USD 667,529.73 |  | USD 1,483,399.39 |  |
| Variable overheads 25% | USD 496,111.30 |  | USD 744,166.96 |  | USD 1,653,704.35 |  |
| **TOTAL RECURRENT COST IN USD** | **USD 2,480,556.52** | **0.66** | **USD 3,720,834.78** | **1.00** | **USD 8,268,521.73** | **2.20** |
| **Utilization 2.00** |  |  |  |  |  |  |
| Consultation costs | USD 878,669.53 |  | USD 1,318,004.30 |  | USD 2,928,898.44 |  |
| Drug costs | USD 5,279,032.06 |  | USD 7,918,548.09 |  | USD 17,596,773.54 |  |
| Human resources | USD 1,780,079.27 |  | USD 2,670,118.90 |  | USD 5,933,597.56 |  |
| Variable overheads 25% | USD 1,984,445.22 |  | USD 2,976,667.82 |  | USD 6,614,817.39 |  |
| **TOTAL RECURRENT COST IN USD** | **USD 9,922,226.08** | **2.64** | **USD 14,883,339.12** | **3.96** | **USD 33,074,086.93** | **8.80** |

**Table S2** presents the results from scenario analysis II, applying the highest recurrent cost of USD 5.00 per consultation. The annual costs would be USD 1,614,197.26 at the national level, assuming a target population of 6% and curative contacts per person per year of 0.25. Expanding the target population to 20% and the curative contacts per person per year to 0.50 would result in a cost of USD 10,761,315.10, equivalent to 2.86 % of Burkina Faso's healthcare budget in 2019.

**Table S2.** Scenario analysis II. Cost and budget impact estimate applying different targeting thresholds and population coverage.

| **Cost category** | **Base case: Targeting threshold 6% of the population:1,290,611** | **% of the healthcare budget** | **Medium assumption scenario: Targeting threshold 9% of the population: 1,935,916** | **% of the healthcare budget** | **High assumption scenario Targeting threshold 20% of the population: 4,302,036** | **% of the healthcare budget** |
| --- | --- | --- | --- | --- | --- | --- |
| **Utilization 0.25** |  |  |  |  |  |  |
| Consultation costs | USD 109,833.69 |  | USD 164,750.54 |  | USD 366,112.31 |  |
| Drug costs | USD 959,014.21 |  | USD 1,438,521.32 |  | USD 3,196,714.04 |  |
| Human resources | USD 222,509.91 |  | USD 333,764.86 |  | USD 741,699.70 |  |
| Variable overheads 25% | USD 322,839.45 |  | USD 484,259.18 |  | USD 1,076,131.51 |  |
| **TOTAL RECURRENT COST IN USD** | **USD 1,614,197.26** | **0.43** | **USD 2,421,295.90** | **0.64** | **USD 5,380,657.55** | **1.43** |
| **Utilization 0.50** |  |  |  |  |  |  |
| Consultation costs | USD 219,667.38 |  | USD 329,501.07 |  | USD 732,224.61 |  |
| Drug costs | USD 1,918,028.42 |  | USD 2,877,042.64 |  | USD 6,393,428.08 |  |
| Human resources | USD 445,019.82 |  | USD 667,529.73 |  | USD 1,483,399.39 |  |
| Variable overheads 25% | USD 645,678.91 |  | USD 968,518.36 |  | USD 2,152,263.02 |  |
| **TOTAL RECURRENT COST IN USD** | **USD 3,228,394.53** | **0.86** | **USD 4,842,591.79** | **1.29** | **USD 10,761,315.10** | **2.86** |
| **Utilization 2.00** |  |  |  |  |  |  |
| Consultation costs | USD 878,669.53 |  | USD 1,318,004.30 |  | USD 2,928,898.44 |  |
| Drug costs | USD 7,672,113.69 |  | USD 11,508,170.54 |  | USD 25,573,712.31 |  |
| Human resources | USD 1,780,079.27 |  | USD 2,670,118.90 |  | USD 5,933,597.56 |  |
| Variable overheads 25% | USD 2,582,715.62 |  | USD 3,874,073.44 |  | USD 8,609,052.08 |  |
| **TOTAL RECURRENT COST IN USD** | **USD 12,913,578.12** | **3.44** | **USD 19,370,367.18** | **5.15** | **USD 43,045,260.39** | **11.45** |
